# The Role of Streptococcal Cell-Envelope Proteases in Bacterial Evasion of the Innate Immune System

**DOI:** 10.1159/000516956

**Published:** 2021-10-14

**Authors:** Sophie McKenna, Kristin Krohn Huse, Sean Giblin, Max Pearson, Mohammed Said Majid Al Shibar, Shiranee Sriskandan, Stephen Matthews, James Edward Pease

**Affiliations:** ^a^Department of Life Sciences, Imperial College London, London, United Kingdom; ^b^Department of Infectious Disease, Imperial College London, London, United Kingdom; ^c^National Heart and Lung Institute, Imperial College London, London, United Kingdom

**Keywords:** Bacterial infection, Cell-envelope protease, Chemoattractants, Complement system, Streptococcus

## Abstract

Bacteria possess the ability to evolve varied and ingenious strategies to outwit the host immune system, instigating an evolutionary arms race. Proteases are amongst the many weapons employed by bacteria, which specifically cleave and neutralize key signalling molecules required for a coordinated immune response. In this article, we focus on a family of S8 subtilisin-like serine proteases expressed as cell-envelope proteases (CEPs) by group A and group B streptococci. Two of these proteases known as *Streptococcus pyogenes* CEP (SpyCEP) and C5a peptidase cleave the chemokine CXCL8 and the complement fragment C5a, respectively. Both CXCL8 and C5a are potent neutrophil-recruiting chemokines, and by neutralizing their activity, streptococci evade a key defence mechanism of innate immunity. We review the mechanisms by which CXCL8 and C5a recruit neutrophils and the characterization of SpyCEP and C5a peptidase, including both in vitro and in vivo studies. Recently described structural insights into the function of this CEP family are also discussed. We conclude by examining the progress of prototypic vaccines incorporating SpyCEP and C5a peptidase in their preparation. Since streptococci-producing SpyCEP and C5a peptidase are responsible for a considerable global disease burden, targeting these proteases by vaccination strategies or by small-molecule antagonists should provide protection from and promote the resolution of streptococcal infections.

## Introduction

Successful host colonization by bacteria is due in part to the evolution of ingenious strategies to evade the innate immune response. If left unchecked, this can result in the initiation of systemic and severe infections. One common strategy amongst diverse bacterial pathogens is the production and secretion of proteases that degrade key components of the immune system. By degrading molecules pivotal to the host response, bacteria are able to evade detection and clearance with a spectrum of clinical consequences [1]. In this article, we focus on a family of S8 subtilisin-like serine proteases expressed by the Gram-positive bacteria *Streptococcus pyogenes* (group A streptococcus [GAS]) and *Streptococcus agalactiae* (group B streptococcus [GBS]) [2]. These proteases are expressed as cell-envelope proteases (CEPs) and selectively abrogate neutrophil recruitment by specifically degrading key chemotactic factors vital for the innate immune response. Severe GAS-related disease presents a considerable human global disease burden that is estimated to result in over 600,000 annual cases of invasive infection, including toxic shock and necrotizing fasciitis [3]. GBS is an emerging human and veterinary pathogen and is the leading cause of early-onset severe infant sepsis. The highly specific activity of this family of streptococcal proteases and their role in combating pathogen clearance underline the critical role that neutrophil chemotactic factors play in response to streptococcal infection.

### Neutrophil Recruitment and Activation in the Innate Immune Response

Neutrophils play a major role in the early response of the innate immune system by neutralizing and phagocytosing bacteria. They are guided to sites of microbial infection by molecules known as chemoattractants, typically small proteins up to 10 kDa in size. Chemoattractants function by binding with high affinity to specific G protein-coupled receptors (GPCRs) on the neutrophil surface, coordinating the directional migration (chemotaxis) towards the source of attractant. Neutrophil chemoattractants can be released or secreted by cells in response to infection (e.g., chemokines) or generated by the cleavage of pre-existing soluble factors (e.g., complement fragments) [4]. Both categories of chemoattractants are substrates for the streptococcal CEPs.

### Complement-Derived Neutrophil Chemoattractants

**Fig. 1:**
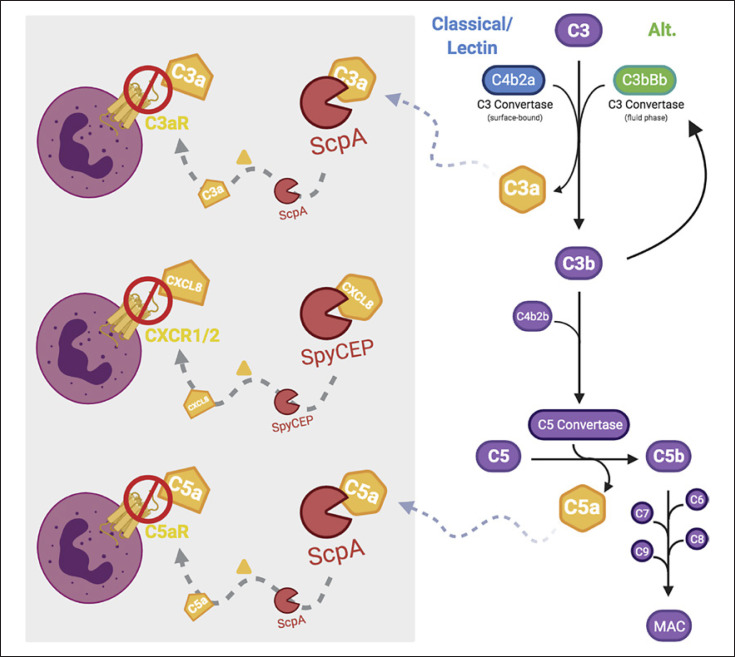
Generation of anaphylatoxins C3a and C5a and subsequent degradations by CEPs. A schematic presentation of the common terminal complement system pathway. The production of anaphylatoxins C3a and C5a, depicted in orange, by the C3 convertases and C5 convertase remains central to the pathways, leading to the formation of the MAC. Pictured in the grey box is the cleavage of chemoattractants by CEPs and the resultant inability to bind and activate their respective receptors. CEPs, cell-envelope proteases; MAC, membrane attack complex; SpyCEP, *Streptococcus pyogenes* CEP. Adapted from Monk et al. [7]. Created with BioRender.com.

The complement pathway is a tightly regulated network of self-perpetuating proteolytic cascades that result in the clearance of pathogens by opsonization, anaphylatoxin release, and formation of the lytic membrane attack complex. Although the membrane attack complex is not known to play a role in the clearance of streptococci, anaphylatoxins C3a and C5a are critical neutrophil chemoattractants, while complement C3b-mediated opsonization is required for neutrophil phagocytosis of streptococci [5]. Both the anaphylatoxins and C3 act as substrates for CEPs. The complement cascade is activated by 3 routes: namely, the classical, lectin, and alternative pathways, which converge on the central complement component and α-macroglobulin family member, C3 [6]. C3 comprises an α chain (111 kDa) and a β chain (75 kDa), linked by multiple disulphide bonds. C3 is modified by the enzyme C3 convertase, which liberates the 9-kDa C3a fragment from the N-terminus of the C3 α-chain, shown in Figure 1. The remaining fragment of C3, known as C3b, contains the 102-kDa α-chain and the 75-kDa β-chain, and is the activated form of C3. The cleavage of C3 into C3b exposes a reactive thioester residue, permitting covalent deposition on the surface of the bacteria. C3b binds the pro-enzyme factor B and is cleaved by factor D to form the enzyme complex C3b2Bb, known as C5a convertase, which cleaves the complement component C5. Cleavage of C5 by C5a convertase releases the N-terminal 10.4-kDa component C5a in a similar manner to the liberation of C3a from C3. Generation of the C3 convertase is controlled by the regulatory protein factor I, a plasma protease that cleaves C3b into the inactive form iC3b. Unlike intact C3b, iC3b is unable to bind factor B and therefore amplify the complement cascade. Both C3b and iC3b function as opsonins, binding to 4 distinct complement receptors (CR1–CR4) expressed by neutrophils and monocytes. Binding to the receptor triggers the phagocytosis of pathogens. CR1 (CD35) binds C3b, while iC3b can bind to CR2 (CD21), CR3 (CD11b/CD18), and CR4 (CD11c/CD18).

**Fig. 2:**
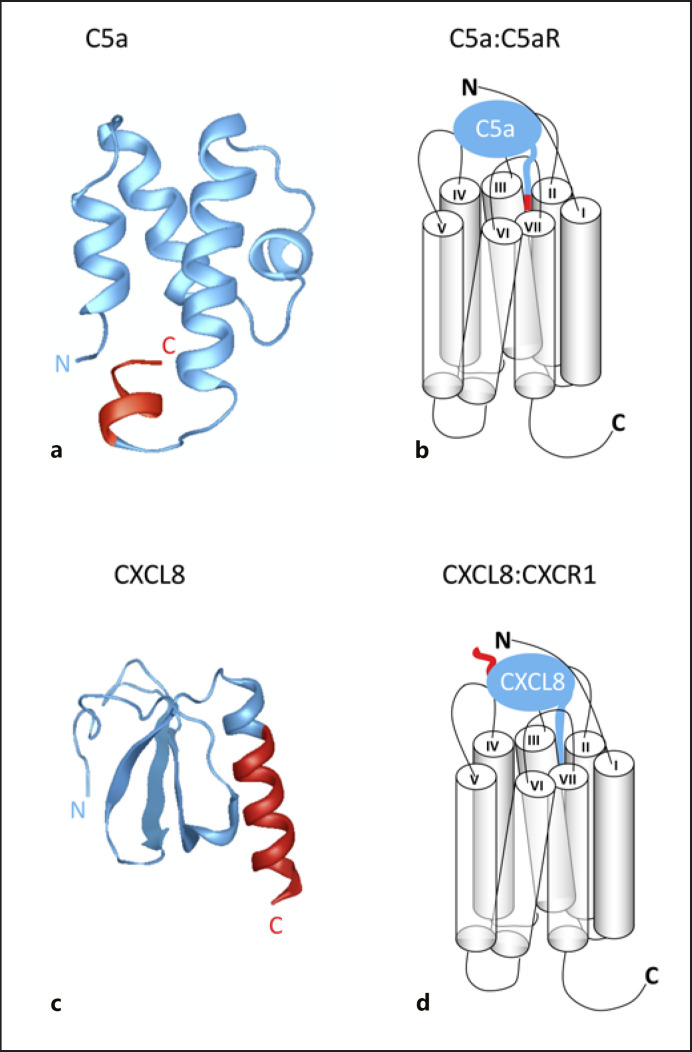
C5a and CXCL8 activate their receptors via distinct two-step models. **a** Cartoon of C5a derived from the PDB file 1KJS with N- and C-termini denoted. **b** Schematic diagram highlighting how the C-terminal portion of C5a inserts into the C5aR helical bundle to induce signalling. **c** Cartoon of CXCL8 derived from the PDB file 1IKL with N- and C-termini denoted. **d** Schematic diagram highlighting how the N-terminal portion of CXCL8 inserts into the C5aR helical bundle to induce signalling. In all panels, the portion of the chemoattractant coloured red marks the piece cleaved from the main body of the chemoattractant (blue) by the CEPs. CEPs, cell-envelope proteases. Generated with Protean 3D^TM^ version 17.0.1, DNASTAR, Madison, WI.

The structures of C3a and C5a anaphylatoxins have been solved by NMR [4] and comprise a 4-α-helical bundle, shown in Figure 2a. C3a and C5a function by binding and activating specific GPCRs known as the C3a receptor (C3aR) and the C5a receptor (C5aR) (reviewed in [7, 8]). Both ligands bind to their receptors in a two-step model in which the N-termini of the receptors tether the anaphylatoxins. This orientates them so that the C-terminal-most residue (arginine) is able to insert into the helical bundle of the GPCR and stabilize an active conformation. This results in the activation of G proteins, as shown in Figure 2b. A recent crystal structure of the C5aR in complex with the small-molecule antagonist PMX53 [9] validated earlier mutagenesis and modelling efforts, suggesting that the C-terminal R75 of C5a contacts D282 of C5aR [10, 11]. It will be seen that the C-termini of both C3a and C5a, which are targets of the streptococcal CEPs, are critical for anaphylatoxin function.

### Neutrophil-Specific Chemokines

The major neutrophil-recruiting chemokines are a subset of the CXC chemokine family possessing an N-terminal glutamate-leucine-arginine (ELR) motif, termed ELR^+^ chemokines [12]. These are exemplified by CXCL8/interleukin-8, the first chemokine to have its structure solved in 1989 [13]. All chemokines contain a conserved tertiary structure comprising a disordered N-terminal signalling domain and a structured core domain consisting of an N-loop, which contains a 3-stranded antiparallel β-sheet, and a C-terminal helix as shown in Figure 2c. Activation of the chemokine receptors follows a two-step model that is analogous to the anaphylatoxin receptors. However, unlike C3a and C5a, tethering by the receptor is followed by the N-terminus of the chemokine inserting into the helical bundle of the chemokine receptor. This drives a conformational change that induces intracellular signalling, shown in Figure 2d. The two-step model was derived by early mutagenesis studies of CXCL8 and its receptors CXCR1 and CXCR2. These studies identified a role for the CXCL8 N-terminus in receptor activation following ligand binding and recognition [14, 15]. Recently, this was validated by a cryo-EM-derived structure of CXCR2 in complex with CXCL8 and G protein [16]. Unlike the anaphylatoxins, therefore, the C-terminus of chemokines lacks apparent specific activity.

## Targeting of Neutrophil Chemoattractants by Streptococcal CEPs

### The C5a Peptidases of GAS, GBS, and Other Streptococci

The first streptococcal CEP discovered was C5a peptidase from GAS, known as ScpA. ScpA cleaves anaphylatoxin C5a [17, 18], targeting the His-Lys peptide bond that is not accessible prior to processing of C5 [19]. Lynskey et al. [20] showed that ScpA cleaves C3a at a site analogous to that in C5a, effectively removing the C-terminus. Given the role of the C-terminus in anaphylatoxin signalling (Fig. 2a–b), it is not surprising that cleavage results in the inactivation of both C3a and C5a.

**Fig. 3:**
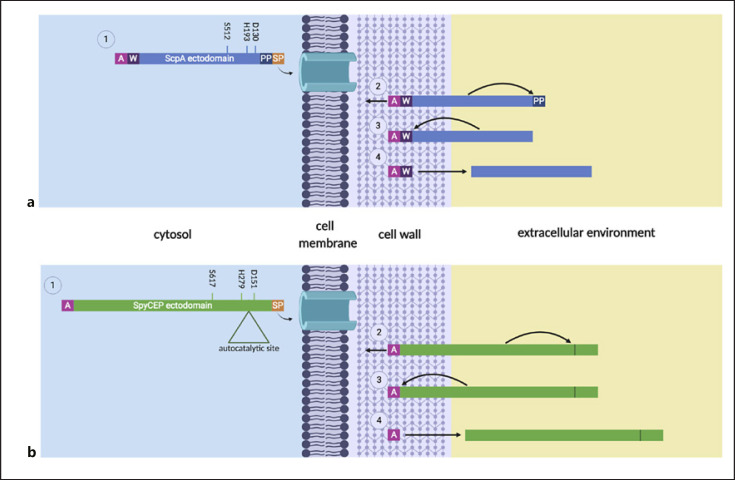
Model for ScpA (**a**) and SpyCEP (**b**) export, processing, and maturation. a. ScpA: (1) The signal peptide (Sp) of ScpA is recognized, mediating protein translocation across the membrane, and removed. (2) Post-translocation, the anchoring domain (A) mediates covalent cell wall attachment through a sortase-dependent mechanism and ScpA autocatalytically removes its prosequence (PP), activating itself. (3) and (4) ScpA is liberated from the cell wall by either an uncharacterized enzymatic route or autocatalysis of the cell wall spanning region (W). b. SpyCEP: (1) The Sp of SpyCEP is recognized, mediating protein translocation across the membrane, and removed. (2) Post-translocation, the anchoring domain (A) mediates covalent cell wall attachment through a sortase-dependent mechanism and SpyCEP autocatalytically liberates the N- and C-terminal domains, producing the heterodimeric mature enzyme. (3) and (4) SpyCEP is liberated from the cell wall by either autocatalysis or an uncharacterized enzymatic route. SpyCEP, *Streptococcus pyogenes* cell-envelope protease. Created with BioRender.com.

ScpA is 1,167 amino acids in length and is initially produced as a precursor; the N-terminal signal peptide directs ScpA to the bacterial cell wall for secretion into the extracellular environment and is autocatalytically removed during this process, while an LPXTG motif at the C-terminus mediates attachment to the streptococcal cell wall through sortase-mediated processing (Fig. 3a) [18, 19]. Further autocatalytic processing generates catalytically active ScpA through the removal of the pro-sequence [21]. The exact N-terminal residue after cleavage has not been identified, so the length of the pro-sequence remains unclear, but crystallographic analysis has shown that the mature and active form of ScpA starts at residue 97 [22]. The role of ScpA in GAS infection is emerging. Early in vivo experiments showed that the expression of ScpA by GAS strains injected intraperitoneally led to delayed accumulation of neutrophils into the peritoneum of mice but did not affect overall GAS virulence [23]. However, intranasal immunization of mice with ScpA led to delayed GAS clearance, suggesting a greater role for ScpA in noninvasive than invasive infection [24]. There are structural differences between mouse and human anaphylatoxins. ScpA-mediated cleavage of human C5a and C3a is now known to be rapid in contrast to cleavage of murine homologues, potentially offering some explanation for results in mice [20]. Indeed, recent studies using a soft tissue model of infection confirmed that any activity of ScpA in mice might be complement independent. Expression of ScpA reduced clearance of GAS in mice lacking both C3 and C5 [20]. It seems possible that ScpA contributes to virulence by promoting GAS adhesion to epithelial and endothelial cells, in a manner independent of catalytic activity, at least in mice [20]. Notwithstanding findings in mice, ScpA-mediated C3a and C5a cleavage leads to a marked reduction in human neutrophil activation and chemotaxis, while cleavage of C3 leads to reduction in opsonization and phagocytosis of GAS. Taken together, ScpA likely plays a key role in human infection that cannot be fully modelled in mice.

Consistent with a role as an adhesin, the homologue of ScpA in GBS, ScpB, possesses adhesive properties in addition to C5a cleavage activity. A phage-display approach identified ScpB as binding fibronectin with high affinity, which was confirmed with a GBS mutant deficient in ScpB expression which showed 50% less binding to fibronectin than its wild-type parent strain [25]. A follow-up study showed the affinity of ScpB to be in the nanomolar range, supportive of a role in virulence [26]. Both studies explain an earlier observation that an anti-ScpB serum raised in rabbits could block the invasion of the human lung epithelial cell line A549 in vitro [27]. Although GBS are not known to replicate within lung epithelial cells, they are able to survive within these cells and their entry via attachment to endogenous fibronectin may represent a mechanism through which GBS can traverse mucosal membranes [27]. As such, ScpB has been termed an “invasin.”

In GAS, the gene ScpA is situated within an operon encoding a number of anti-immune response virulence factors such as M protein and the streptococcal inhibitor of complement, the expression of which is positively regulated by the global transcriptional regulator Mga [28]. In contrast, evidence suggests that ScpB is predominantly regulated by the 2-component CsrRS regulator in GBS, which is also known as CovR/S in GAS [29]. Deactivating mutations in both CsrR and CsrS led to de-repression of ScpB transcription and increased expression [29]. However, whether this effect is due to a direct interaction between CsrRS and *scpB* remains unclear.

In addition to being a leading human pathogen associated with neonatal sepsis and meningitis, GBS is also a major causative agent of bovine mastitis. ScpB and Lmb (laminin-binding protein) are found on a transposon, flanked by insertion sequences in GBS, and these genes show high sequence identity to the genes in GAS, suggesting acquisition by horizontal gene transfer [30]. Intriguingly, this transposon is absent from many bovine isolates of GBS [31]. In vitro analyses of an Scp reporter gene construct found that the expression of ScpB and Lmb is correlated with exposure to increasing levels of human, but not foetal calf serum in strains possessing the transposon [32].

Homologues of C5a peptidase are found in other streptococcal species. A gene encoding a subtilisin-like serine protease with domain architecture similarity to C5a peptidase was recently discovered in the emerging zoonotic pathogen, *Streptococcus suis* [33]. Increased expression of this gene, SSU1773, was identified in the blood, joints, and hearts of infected pigs that were infected with a highly virulent strain of the pathogen [33] and is implicated in adhesion to porcine cells in vitro as it was identified during a transposon-directed insertion sequencing screen of *S. suis* genes involved in adhesion and colonization of pig nasal epithelium [34]. *Streptococcus equi* and *Streptococcus zooepidemicus* are predicted to encode CEPs named ScpZ whose substrate is C5a [35, 36], while *Streptococcus iniae* is a pathogen associated with finfish aquaculture and has been shown to encode a C5a peptidase known as ScpI [37]. PCR analysis of virulence factors coupled with *emm* typing found that the vast majority (99.2%) of 246 isolates of *Streptococcus dysgalactiae* subspecies *equisimilis* (SDSE) harboured the gene encoding ScpA [38]. SDSE is a beta-haemolytic streptococcal species that causes similar disease presentations to GAS. Table 1 summarizes functionally related streptococcal CEPs shown to inactivate chemotactic factors.

### SpyCEP, the CXCL8-Cleaving Serine Protease of GAS

The *S. pyogenes* CEP (SpyCEP) was originally identified by its ability to cleave human CXCL8, after it was noted that lethal necrotizing GAS infections were characterized by poor neutrophil recruitment to sites of infection despite the significant bacterial burden [39, 40]. SpyCEP is a 180-kDa heterodimer comprising 2 non-covalently linked polypeptide fragments derived from autocatalytic processing [41]. N- and C-terminal fragments, 30 and 150 kDa, respectively, both contribute residues to the catalytic triad (D151, H279, and S617) and can be produced separately and recombined to produce a functional enzyme [42]. Similar to ScpA, SpyCEP is secreted via an N-terminal signal peptide and anchored to the bacterial cell wall by the C-terminal LPXTG cell wall-anchoring motif (Fig. 3b) [40]. SpyCEP is subsequently liberated from the cell wall by an undefined mechanism and found free in culture [43].

In addition to CXCL8, SpyCEP cleaves all neutrophil-specific chemokines that possess an ELR motif, namely, CXCL1, CXCL2, CXCL3, CXCL5, CXCL6, and CXCL7, thereby inhibiting the recruitment of neutrophils to sites of infection and inflammation [44, 45]. Evolution and conservation of the gene encoding SpyCEP are consistent with a strong selective pressure that favours subversion of neutrophil-specific chemokines. As the major neutrophil-specific chemokine, it seems likely that CXCL8 is the main substrate of SpyCEP, while other ELR^+^ chemokines serve as inadvertent substrates due to shared structural homology [45]. In the case of CXCL8, SpyCEP cleaves the peptide bond between Q59 and R60, resulting in the production of a 59-amino acid N-terminal fragment, and a 13-amino acid C-terminal fragment that corresponds to the C-terminal α-helix. Despite the high level of amino acid sequence identity between ELR^+^ CXC chemokines, the cleavage sites of CXCL1, CXCL2, CXCL6, and CXCL8 vary in their primary sequence but exhibit high secondary structural homology, wherein a C-terminal α-helix is liberated by SpyCEP in each case [46]. This is perhaps curious since the C-terminus of the CXC chemokines has not been considered to play a major role in chemokine signalling and might not, alone, be predicted to influence receptor ligation and chemokine activity.

### Chemokine Inactivation by SpyCEP

Cell membranes are typically decorated with a negatively charged glycocalyx comprising glycosaminoglycans (GAGs), repeating negatively charged disaccharide units. The C-termini of chemokines are typically positively charged and have been defined in many instances as a GAG-binding site. This is certainly true for CXCL8, which possesses a cluster of positively charged residues within the C-terminus (notably R60 and K67) that are critical for supporting the electrostatic interactions, which drive GAG binding [45, 46, 47, 48, 49]. GAGs are essential for the proper functioning of chemokines in vivo [47] and exhibit changes in their structure and localization following tissue injury and inflammation (reviewed in Ref. [48]). While GAGs display diverse physiological roles that support tissue homeostasis, their interactions with chemokines on the vascular endothelium support haptotactic cell migration by establishing stable cell-surface chemokine gradients that are detected by leukocytes in the peripheral circulation [49]. For example, chemokines produced in response to a tissue infection have to be translocated to the endothelial surface to be visible to neutrophils in the periphery. Without the physical interaction with endothelial GAGs, the chemokine would be washed away by the blood flow within the vessel, rather acting being concentrated close to the site of infection. Furthermore, the ability of chemokines to form oligomers on GAGs allows concentrated “depots” of chemokines to be generated to enhance leukocyte recruitment. Although mutant obligate monomeric forms of chemokines have been shown to be active in in vitro chemotaxis assays, such forms are often inactive in vivo, highlighting the importance of oligomer formation [47]. In the case of CXCL8, GAG binding via the C-terminal α-helix facilitates both the translocation of the chemokine to the luminal endothelial surface [50] and the oligomerization of CXCL8 into dimeric and higher order species [51]. Since SpyCEP cleaves the C-terminal GAG-binding motif from CXCL8, it was originally postulated that the lack of neutrophils observed in post-mortem tissues from a patient with invasive GAS infection was due to a lack of CXCL8 on the endothelial surfaces, able to induce their arrest and transmigration from the peripheral circulation [40, 52]. However, this hypothesis did not explain the manifest loss of chemotactic activity exhibited in vitro [40].

Recent work from Goldblatt et al. [45] has extended these observations to put forward a role for neutrophil GAGs in CXCL8 signalling. Cleavage of CXCL8 by SpyCEP was shown to render the chemokine unable to bind CXCR1 and CXCR2 in vitro, with a subsequent loss of all downstream signalling events including chemotaxis. SpyCEP cleavage of CXCL8 was also shown to ablate heparin binding, and subsequent glycanase treatment to remove cell-surface GAGs from neutrophils was shown to abolish CXCL8-induced activation of neutrophils in vitro. Collectively, these data support the hypothesis that chemokine binding to neutrophil-bound GAGs is required for the subsequent activation of CXCR1 and CXCR2. These interactions expose an inherent vulnerability in the initiation of innate immunity, which is exploited by SpyCEP to inactivate CXCL8 in vivo [52].

The removal of the GAG-binding region of CXCL8 by SpyCEP also introduces the possibility that SpyCEP subverts host immunity by interfering with the process of chemokine oligomerization. Neutrophil pericellular GAGs comprising the glycocalyx sequester local soluble chemokines in multiple degrees of oligomerization, to sample the chemokine gradients present at the leading edge of the neutrophil. In situ, CXCL8 can form monomeric and dimeric variants, with the dimer exhibiting reduced activation of CXCR1 in chemotaxis assays relative to the monomeric form [53]. While both monomeric and dimeric CXCL8 bind GAGs, they are unable to activate CXCR1 or CXCR2 while bound [54]. It is thought that neutrophil cell surface GAGs serve to increase local concentrations of chemokines in the vicinity of their cognate receptors to improve the chemokine gradient sampling efficiency. Therefore, in addition to its impact on reducing CXCR1/CXCR2 signalling, SpyCEP may also disrupt CXCL8 oligomerization and in turn, reduce CXCL8 dimer dissociation into its receptor-activating monomeric subunits.

### SpyCEP Impact on Pathogenesis

In addition to human ELR^+^ CXC chemokines, SpyCEP cleaves the murine CXCL1 and CXCL2 homologues MIP-2 and KC, which signal via mCXCR2 and play a significant role in recruiting neutrophils to the sites of infection in mice [40, 55, 56, 57]. Several in vivo experimental studies in mice using strains that differed in SpyCEP gene expression have demonstrated a role for SpyCEP in soft tissue dermonecrosis [44, 46, 55, 58, 59]. Although some studies have demonstrated paradoxical results [46, 58], discrepancies may arise due to differences in models, strains, and the possibility of regulatory gene mutations. Several in vivo studies have demonstrated that SpyCEP expression reduces the levels of murine chemokines in either tissue or serum while retarding neutrophil influx. Interestingly, SpyCEP expression confers a lethal phenotype to the otherwise innocuous *Lactococcus lactis* [46], with an increased bacterial burden and dissemination observed in a SpyCEP-dependent manner. A recent report showed that *S. pyogenes* can utilize draining lymph nodes to metastasize to distant sites following intramuscular injection of *S. pyogenes* into mouse hind limbs [60]. The same study also found a role for SpyCEP in limiting neutrophil recruitment to lymph nodes, thereby promoting survival of GAS and underlining a role for CXC chemokines in leukocyte recruitment to lymph nodes. The implication of the findings from both heterologous expression studies and mutagenesis in *S. pyogenes* is that SpyCEP not only retards neutrophil recruitment but also impairs neutrophil killing of bacteria, which is consistent with previous reports demonstrating that SpyCEP protects *S. pyogenes* from neutrophil killing, in part due to the inhibition of chemokine-induced neutrophil extracellular traps [59].

The SpyCEP gene, *cepA* (also annotated as scpC/prtS), is found ubiquitously in all isolates of GAS; however, the expression of SpyCEP is highly variable between clinical isolates, with invasive blood isolates expressing higher levels of SpyCEP than pharyngeal isolates [40, 61]. In a clinical invasive disease, high SpyCEP expression is associated with disease severity and poor patient outcome [61]. SpyCEP is under the repression of CovR/S (also known as csrRS), the two-component gene regulator that controls approximately 15% of the GAS genome [61, 62, 63]. Naturally occurring mutations at this regulatory locus have been reported to dramatically upregulate SpyCEP expression and contribute to disease severity [40, 61]. Mutations in CovR/S are proposed to occur at the time *S. pyogenes* transitions from a pharyngeal to a more invasive state and may therefore underlie the observed dichotomy in SpyCEP production between pharyngeal and blood culture isolates.

## Other CEPs of Pathogenic Streptococci

The family of CEPs in pathogenic streptococci has orthologues in other lactic bacteria, namely, the lactococci and *Streptococcus thermophilu*s (prtS), where almost all have a nutritional role in cleavage of casein [64, 65, 66]. Along with orthologues to SpyCEP (e.g., SpyCEP in *S. dysgalactiae* subsp*. equisimilis*, SeCEP in *S. equi*, and SzoCEP in *S. zooepidemicus*) and C5a peptidase, other CEPs have been discovered in pathogenic streptococci. The functions of some of these proteases are yet to be elucidated; however, like SpyCEP and C5a peptidase, most target effectors of the host innate immune system.

### PrtA

PrtA was discovered in *Streptococcus pneumoniae* by screening convalescent-phase serum and was identified as a serine protease containing the histidine, aspartate, and serine catalytic triad [64]. Unlike SpyCEP and C5a peptidase, which maintain a high degree of sequence identity between strains of the same species, PrtA displays a surprising amount of diversity between clinical strains of *S. pneumoniae*, particularly within the central region of the protein [67]. The exact function of PrtA has not been established, but in vivo evidence is emerging of its role as a virulence factor. Loss of the gene does not impact colonization of the murine nasopharynx, although it does result in attenuated virulence and lower recovery of bacteria from the lung and blood of infected mice [68]. Furthermore, mice infected intraperitoneally with a prtA knockout *S. pneumoniae* strain showed greater survival compared to mice infected with the prtA-expressing WT parent strain [67]. Interestingly, it was shown that PrtA cleaves human apolactoferrin, releasing lactoferrin-like peptides with bactericidal activity. Therefore, somewhat paradoxically, expression of *prtA* may lead to decreased bacterial survival [69].

PrtA is transcriptionally regulated by PsaR in *S. pneumoniae* [70], and while the function of PrtA is unknown, its regulation indicates a role in the progression from nasopharyngeal carriage to more invasive disease. The PsaR regulon is highly upregulated in the presence of nickel, cobalt, and zinc ions and repressed in the presence of magnesium ions [71, 72, 73]. Responding to changes in metal ions is thought to be important for adapting to the change in environment from the nasopharynx to lungs. Interestingly, in vivo expression of *prtA* is increased in blood compared to the nasopharynx of infected mice [68]. Clearly, there is much more to learn about PrtA, notably its main function and the role, if any, of diversity within Prt and virulence.

### CspA and SspA

CspA was first identified in a GBS blood isolate from a case of neonatal sepsis. While it was found to share some homology with C5a peptidase and caseinases from lactic acid bacteria, it does not cleave either C5a or casein [74]. At first, fibrinogen was the only known substrate of CspA, but its similarity to other immunomodulating proteases, coupled with the attenuated virulence of a CspA knockout GBS strain in a neonatal rat infection model, suggested that CspA may also serve a role in pathogenesis [74]. CspA was subsequently found in in vitro assays to cleave the ELR^+^ CXC chemokines CXCL1, CXCL2, CXCL3, CXCL6, and CXCL7, but not CXCL8 [75]. No cleavage of CC chemokines was shown, although it was not possible for the authors to demonstrate cleavage was not occurring at the extreme C- or N-termini of the proteins [75].

Investigation into the MtaR regulator of GBS, which is required for virulence, showed that CspA was one of the 11 genes downregulated in an MtaR mutant [76], further suggesting a role in GBS infection. Although initially thought to only be located on the cell wall [74], it was later shown that CspA includes a second site for autocatalytic cleavage near the cell wall-anchoring domain, which leads to the release of a mature peptide from the cell wall [75, 77]; however, immunofluorescence staining of GBS has established that CspA maintains a punctate distribution on the cell wall [78].

A homologue of CspA, the serine protease SspA, was discovered in *S. suis*, by screening both the convalescent-phase serum [79] and a transposon mutant library for isolates deficient in cleavage of a chromogenic substrate specific for chymotrypsin-like proteases [80]. SspA possesses a His-Asp-Ser catalytic triad and shares the greatest degree of sequence identity with PrtS of *S. thermophilus* (95.9%) and CspA (49.5%) [80]. Mutants deficient in SspA exhibit delayed growth and decreased survival in whole human blood, while inducing milder clinical signs and significantly decreased mortality in mice following intraperitoneal infection [80]. SspA degrades gelatin [81] and, unlike CspA, degrades CCL5 [82]. A further immunomodulatory role for SspA was uncovered following incubation of stimulated THP-1-derived macrophages with active and heat-inactivated recombinant SspA. This in vitro study showed that regardless of proteolytic activity, SspA induced IL-1β, IL-6, TNF-α, CXCL8, and CCL5 release [82]. An inverse relationship between SspA and CCL5 concentration was observed, as although SspA induced the secretion of CCL5, high concentrations of exogenous recombinant SspA resulted in the degradation of de novo secreted CCL5, while, conversely, low concentrations of SspA led to increased concentrations of CCL5 [82]. Induction of pro-inflammatory cytokines points to another strategy of immunomodulation by bacterial serine proteases. Unlike the secretion of other streptococcal CEPs, evidence suggests that SspA secretion is dependent on a type-4 secretion system (T4SS) encoded by the 89K pathogenicity island of *S. suis* [83]. However, detectable amounts of SspA were found in the growth supernatant of the T4SS knockout strain, and previous work showed that SspA was only present on the cell wall of *S. suis* but not in the supernatant [79]. Another CspA homologue, SFP (subtilase-family protein), was identified in *S. pneumoniae*; however, its role in virulence has yet to be determined [84]. Given that *S. suis* is a major pathogen of pigs worldwide, we expect that this CEP will continue to be the focus of future research.

## Sequence and Structural Homology within Bacterial CEPs

Subtilisin-like serine proteases, termed *subtilases*, are a diverse family found in bacteria, archaea, yeast, fungi, and some eukaryotes [2, 64]. Subtilases are typically extracellular and exhibit low sequence homology and diverse functionality, spanning nutrient scavenging and precursor processing for immune evasion [65]. These proteins are characterized by a multi-domain architecture extending over 1,000 residues, typically comprising an N-terminal signal peptide for export and a complementary C-terminal-anchoring motif, pre-propeptide (removed by autocatalysis), subtilisin-like catalytic domain with an inserted protease-associated (PA) domain, with heterogeneity observed in the composition and number of C-terminal domains. The protease domain encompasses the aspartate-histidine-serine catalytic triad, with the highest sequence conservation observed proximal to the active site, but variation in these residues can be observed [66].

**Fig. 4:**
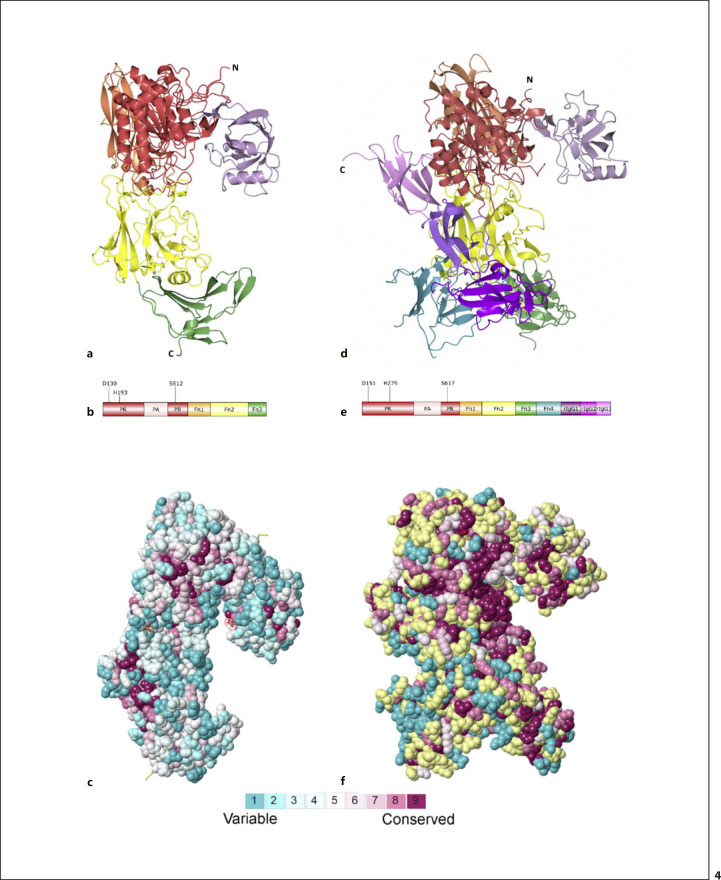
Structures of ScpA and SpyCEP. Structures of wild-type ScpA (3EIF) (**a**) and SpyCEP (5XYR) (**d**), coloured according to the domain architecture shown in (**b, e**), respectively. ConSurf analysis of ScpA (**c**) and SpyCEP (**f**). Sequences of the most closely related C5a and chemokine degrading CEPs were analysed to highlight regions of sequence conservation, then mapped onto the structures, and visualized according to the key. Regions that could not be analysed due to insufficient homologous data are shown in yellow. SpyCEP, *Streptococcus pyogenes* cell-envelope protease. Created with PyMol.

Streptococcal CEPs are members of the S8 family of subtilases and share a common architecture. This comprises a YSIRK signal peptide and a C-terminal LPXTG-anchoring motif, essential for sortase-dependent anchoring and functionality of the protease [85] consistent with cell wall-anchored proteins from Gram-positive bacteria. Bacterial CEPs also contain an S8 catalytic domain interrupted by a PA domain, fibronectin, and immunoglobin-like C-terminal domains and are defined by autocatalytic processing during maturation. Crystal structures have been solved for 3 streptococcal CEPs, SpyCEP, ScpA, and ScpB. The mature and active form of ScpA spans residues 97–1,032, encompassing the catalytic domain with the inserted PA domain followed by 3 fibronectin type III domains (Fn1–Fn3 domains) (Fig. 4a) [22]. The catalytic triad (D130, H193, and S512) is localized in the catalytic domain (Fig. 4b) and is consistent with other characterized S8 subtilases [21, 86]. SpyCEP comprises 9 distinct domains (Fig. 4d, e), where the first 5 domains, catalytic domain with the inserted PA followed by 3 fibronectin type III domains (Fn1–Fn3 domains), are vital for catalytic activity and homologous with the domain organization in ScpA [87]. The remaining 4 domains extend beyond the core and encompass a fibronectin/Indian hedgehog protein (Fn4) domain and 3 reverse-Ig folds.

ScpA and ScpB share 98% sequence identity and domain organization but exhibit a surprising level of structural variance. Alignment of all Cα positions on ScpA and ScpB produces a root mean squared difference of 4.7 Å, owing to differences in the orientation but not fold of the PA and Fn1–Fn3 domains and regions within the catalytic domain [22]. However, the ScpB structure is catalytically inactive as it was produced as a shorter construct, impeding autocatalytic processing vital for protein maturation. This was proposed as the cause of the structural differences by Kagawa et al. [22] and is in line with structural variance observed in wild-type and mutants of SpyCEP [87, 88].

**Fig. 5:**
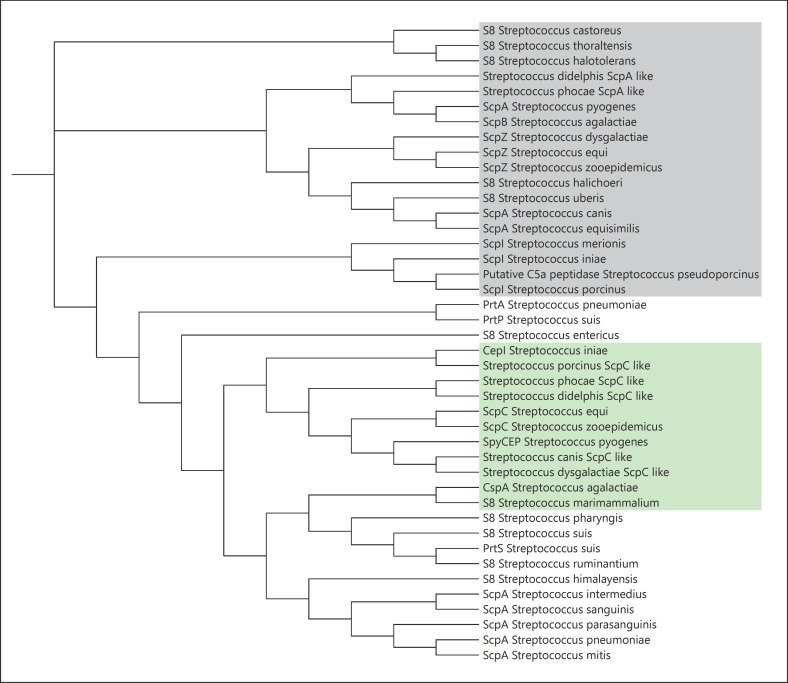
Phylogenetic tree of streptococcal CEPs. ConSurf analysis generated a scored list of related sequences for SpyCEP (5XYR), ScpA (3EIF), and ScpB (1XF1) [22, 87]. The 15 most related sequences were taken, with duplicates removed, for each protein and a phylogenetic tree generated using Clustal Omega [85]. Phylogenetic data were visualized using the Interactive Tree Of Life [88]. Sequences were blasted and denoted ScpA (grey shading) or ScpC (green shading) like owing to their homology (>50%) with C5 peptidase (ScpA) or SpyCEP (ScpC), respectively. CEPs, cell-envelope proteases; SpyCEP, *Streptococcus pyogenes* CEP.

Large-scale genome sequencing has highlighted the prevalence and wide distribution of streptococcal S8 subtilases, whereby function is inferred from the annotation of the S8 catalytic region and homology with ScpA/B or SpyCEP. We carried out phylogenetic analysis of streptococcal CEPs [89, 90, 91, 92], most closely related to SpyCEP and ScpA/B, and found that there is a clustering of sequences with related and experimentally characterized function, where 2 distinct subtrees, related to C5a and CXC chemokine-degrading functions, are observed (Fig. 5). Although functional assignments cannot be made without experimental confirmation, sequences with homology greater than 45% to either ScpA/ScpB or SpyCEP cluster around these branches and have been highlighted. The retention of CEPs across a wide array of pathogenic streptococci, displayed in only the most closely related subset here, indicates the importance of the function played by these proteins in the establishment of infection.

Sequence homology within the streptococcal CEPs is low, but functionally related proteins exhibit greater homology. SeCEP and SzoCEP from *S. equi* and *S. zooepidemicus*, respectively, share 96% sequence identity and 61–62% with SpyCEP (Table 2). Experimental characterization has shown that they can cleave human and equine CXCL8 [52]. This sequence identity is concentrated in the protease-related region of the sequences, which is unsurprising when shared substrate specificity is considered. ConSurf, a bioinformatic server that calculates the evolutionary conservation of each amino acid in a protein based on phylogenetic links between homologous sequences, was used to analyse the SpyCEP sequence, with highest sequence conservation corelating with an annotated chemokine degradation function [90, 91]. Highest sequence conservation, when mapped onto the structure of SpyCEP, is observed in the core catalytic domains with least conservation observed at sites most distal to the core of the protein (Fig. 4f). Interestingly, the PA domain exhibits patches of high sequence conservation but has been shown to be mobile in molecular dynamics simulations. It has been hypothesized that the PA domain plays a role in substrate recruitment, with this sequence conservation observation indicative of a functional relevance [88].

Comparison of ScpA with closely related C5a degrading CEPs elicits a similar result, with the highest sequence conservation observed in the residues comprising the core of the catalytic domain (Fig. 4c). However, on average, ScpA exhibits less overall sequence conservation than SpyCEP. For example, only the core of the more ordered PA domain is highly conserved in ScpA in comparison with the entire PA domain of SpyCEP. Interestingly, ConSurf identified more related sequences for ScpA than SpyCEP, which could have given rise to the lower overall sequence conservation score, with a wider evolutionary space sampled for ScpA. Moreover, SpyCEP has a higher sequence identity with related CEPs (Table 2), further contributing to the high sequence conservation observed.

Intrinsically disordered regions (IDRs) are a common feature at the extreme N- and C-termini of CEPs, imparting flexibility required for autocatalysis-mediated maturation and a high degree of freedom to a cell wall-anchored protease. These regions are typically missing from crystallographic analysis but have been characterized for SpyCEP using NMR [88]. The N- and C-terminal IDRs present in SpyCEP (residues 34–115 and 1,575–1,613, respectively) are characterized by distinct subsets of disorder, highlighting the functional plasticity imparted by these sequences particularly within the context of a structured protein. The C-terminal IDR is directly upstream of the LPXTG-anchoring motif, exhibits limited structural propensity, and is hypothesized to function as a flexible linker. However, the N-terminal IDR exhibits helical propensity indicative of functional relevance. Moreover, truncation of this region impedes crystallization [93] and inhibits the heterodimerization of autoprocessed SpyCEP. Interestingly, IUPRED analysis of the most closely related sequences to SpyCEP, SeCEP and SzoCEP, highlights the consistent presence of disorder at the extreme N-termini but not the C-termini [94, 95, 96]. The predicted retention of the N-terminal IDR indicates the importance of this region within the CEPs. Further analysis is required to determine functional relevance and the role of disorder within the CEPs, potentially broadening or confining the observations made on SpyCEP.

## Vaccine Development Targeting Pathogenic CEPs

There is a global demand for robust vaccines against pathogenic streptococci. Worldwide *S. pneumoniae* and *S. pyogenes* infections alone account for over 2.1 million deaths per year [97, 98], highlighting the significant healthcare burden and requirement for accessible global vaccines. Developments over the last 2 decades in reverse vaccinology and cell surface proteomics have revealed many novel and immunogenic streptococcal proteins as potential vaccine targets. In a field that is increasingly searching for highly conserved pan-serotype antigens, the CEPs of pathogenic streptococci represent ideal candidates for inclusion in both inter- and intraspecies vaccine designs. In this section, we will discuss the use of CEPs as vaccine components (summarized in Table 3).

### SpyCEP as a Target for GAS Vaccination

SpyCEP has been the target of several GAS vaccine designs. It was initially identified through “reverse vaccinology” as a potentially protective cell wall-associated antigen [99]. Immunization with recombinant SpyCEP conferred a 70% survival rate to CD1 mice challenged with a 90% lethal intranasal dose of a serotype M23 *S. pyogenes*. Subsequently, vaccine studies have shown SpyCEP immunization to induce protease-neutralizing antibodies in mice [52, 100], raising the possibility that vaccine-induced protection resides in the neutralization of virulence as much as opsonic activity.

Turner et al. [52] demonstrated that SpyCEP immunization enhanced protection against *S. pyogenes* dissemination in both intranasal and intramuscular infection models in mice and highlighted the potential of SpyCEP to induce cross-species protection against *S. equi* intramuscular infection. More recently, the combination of SpyCEP with 2 additional conserved, highly expressed, and immunogenic proteins, arginine deaminase and streptolysin O, demonstrated cross-serotype (M1, M6, M12, and M23) protection in CD1 mice, conferring 50–80% survival following intranasal and intramuscular challenge and significantly reducing bacterial growth in a subcutaneous air pouch infection model [101]. This combination vaccine is currently in commercial development, while SpyCEP has also been used in a number of other combination vaccines [100, 102, 103].

SpyCEP has also been used to augment the efficacy of M protein-based vaccines, the historical target of *S. pyogenes* vaccines. An M protein-based, minimal B-cell epitope vaccine conjugated to the diphtheria toxoid, named J8-DT, was shown to be effective against pyoderma in mice, but ineffective against hypervirulent *S. pyogenes* strains that had mutations in covRS and therefore expressed an abundance of virulence factors [104]. The inclusion of SpyCEP, or a 20-amino acid (aa 205–224) minimal epitope, in the J8-DT vaccine effectively protected against these hypervirulent strains [105, 106].

### ScpA as a Target for GAS Vaccination

ScpA was proposed as a universal candidate GAS vaccine almost 30 years ago, to circumvent the problems inherent in vaccinating against GAS, a pathogen with multiple “M” serotypes [19, 107]. Immunization with recombinant ScpA is highly immunogenic in rabbits and mice and can induce cross-serotype (M1, M2, M6, M11, and M49)-neutralizing antibodies and reduce cross-serotype streptococcal colonization [24]. Modern immunoproteomic techniques, including reverse vaccinology and approaches to uncover the anti-GAS-protective antigenic targets of human intravenous immunoglobulin, have identified and confirmed ScpA to be a key protective antigenic target in GAS infection [99, 102]. More recent work has therefore included ScpA in various combination vaccines, such as Spy7 [102] and Combo5 [100], all of which have shown protective effects in different animal models with Combo5 showing protection in rhesus macaques.

Given the ability of ScpA to modulate human neutrophil recruitment and opsonization, it is unsurprising that it has emerged as a key target in *S. pyogenes* vaccine design. Anti-ScpA-specific IgG and secretory IgA immunoglobulins are detectable from convalescent patient samples, with antibodies purified from sera effectively neutralizing ScpA activity [108]. Immunization studies have additionally highlighted ScpA as a pan species vaccine target, demonstrating that specific ScpA-induced antibodies can inhibit protease activity of C5a peptidase from both GAS and GBS [109]. Intranasal immunization with ScpA adjuvanted with cholera toxin significantly reduced M49 *S. pyogenes* colonization of mouse nasal-associated lymphoid tissue [110]. Additionally, intranasal administration of immunized sera containing specific anti-ScpA immunoglobulin provided passive protection in naive mice. Given the reduced enzymatic functionality of ScpA in mice, it seems likely that the protection afforded may reside in opsonic or anti-adhesin activity; the mechanisms by which immunization confers protection require elucidation.

### ScpB as a Target for GBS Vaccination

As described above, immunization of mice with either ScpA or ScpB induces neutralizing antibodies and can reduce the bacterial burden following a subsequent intranasal challenge with GAS [109]. Similarly, adjuvanted ScpB immunization can reduce lung bacterial burden following a serotype VI GBS intranasal challenge, while ScpB immune antiserum can additionally provide passive cross-protection from both GAS and GBS infections [109, 111].

Vaccine initiatives against GBS are primarily aimed at reducing the risk of infant infections, which are associated with maternal colonization at the time of birth. As GBS can be a normal part of the enteric flora in up to 30% of healthy women, induction of long-term immunity is challenging. A number of strategies have evolved to elicit effective and longer lasting anti-GBS immunity including encapsulation of ScpB in a biodegradable polymer and combination with a GBS surface-exposed lipoprotein [113, 114, 115]. While all have shown some promise in different models of infection, none have yet reached clinical evaluation.

### Streptococcus suis

*Streptococcus suis* is a global swine pathogen and a potent zoonotic agent. While zoonotic infections are rare, *S. suis* can cause meningitis, sepsis, and death in humans. This burden is particularly high in Asia, where over 90% of human cases occur [116]. In Southern Vietnam, *S. suis* is the leading causative agent of adult meningitis [117] and responsible for a mortality rate of up to 6% [118]. Historically vaccine designs for *S. suis* have focused on bacterins (attenuated bacteria); however, the field is now shifting towards conserved proteins including the CEP SspA and other proteins to develop cross-serotype protection [119, 120]. Despite these efforts, there is currently no effective commercially available vaccine developed against *S. suis*.

### Streptococcus pneumoniae

Despite the introduction of both the 23-valent polysaccharide (PPV23) vaccine and the 10/13-valent conjugate vaccine (PCV10/13), there is still a large global health burden associated with *S. pneumoniae* infection. Vaccine coverage remains a challenge, however, due to a need to combat non-vaccine serotypes and capsule switching; development of the next generation of protein-based vaccines may require the use of pan-serotype antigenic targets including virulence factors such as the CEPs. The *S. pneumoniae* CEP PrtA, known to cleave apo lactoferrin, can evoke protective immunity in some animal models of infection and could, in combination with other antigens, provide a solution to the problems of serotype specificity in pneumococcal vaccinology as demonstrated in a number of murine models of pneumonia [121, 122, 123].

## Conclusion − Looking Ahead

The CEPs of pathogenic streptococci have an exclusive relationship with their substrates; no other group of enzymes targets the entire family of neutrophil-active CXC chemokines or neutrophil families of chemotactic agents. Beyond the fascinating role that the CEPs play in circumventing host innate immunity, this family of serine proteases may yet have additional functions related to adhesin activity and play an important role in future vaccine development strategies.

The knowledge gained by understanding GAG-chemokine interactions and their interdiction by proteases, such as SpyCEP, raises possibilities for therapeutic development. Potential SpyCEP antagonists modelled on CXCL8 but with reduced CXCR1 and CXCR2 activity have previously been described [14, 15] and could in theory provide adjuvant therapies for more severe invasive streptococcal infections that fail to respond to antibacterial agents alone. However, the ability of CEPs to exhibit class action against entire families of neutrophil chemoattractants raises the possibility that their activities might be harnessed in the treatment of inflammatory disorders − particularly those where CXC chemokines or anaphylatoxins are proven deleterious mediators. Examples of disorders known to be driven by CXCL8 might include inflammatory disorders such as Crohn's disease, COPD, and ARDS; however, caution would be required to ensure impedance of neutrophil recruitment did not render subjects prone to bacterial infections. ScpA has already been developed as a potential anti-inflammatory immune modulator, although clinical application is yet to be demonstrated. As humans often develop antibodies to bacterial enzymes such as ScpA and SpyCEP, such therapies may require better understanding of enzyme activity and development of novel agents that emulate the native bacterial enzymatic action. Of more recent relevance, it has been reported that CXCL8 and C5a are present in abundance in hospitalized patients with COVID-19 [124, 125]. If confirmed to be deleterious, then either SpyCEP or ScpA analogues might be of value either as locally or as systemically delivered reagents in the more severe cases.

## Conflict of Interest Statement

The authors have no conflicts of interest to declare.

## Funding Sources

Our work in this area is supported by the Wellcome Trust Collaborative Award 215539: “Understanding and exploiting Group A streptococcal anti-chemotactic proteases in vaccines for infection” awarded to S.S., S.M., and J.E.P.

## Author Contributions

S.McK., K.K.H., S.G., M.P., M.S.M.A.S., S.S., S.M., and J.E.P. all contributed to the writing of the initial draft of the manuscript and subsequent edits.

## Figures and Tables

**Table 1:** Summary of functionally related chemotactic factors inactivating streptococcal CEPs

Species	Protease	Substrate	Location	References
Group A streptococcus	SpyCEP	CXCL1, CXCL2, CXCL3, CXCL5, CXCL6, CXCL7, and CXCL8	Cell surface and released	[45]
	
	ScpA	C5a, C3, and C3a	Cell surface and released	[20]

Group B streptococcus	CspA	Fibrinogen and other chemokines	Cell surface and released	[75]
	
	ScpB	C5a	Cell surface and released	[27]

*Streptococcus equi*	SeCEP	CXCL8	Cell surface and released	[52]
	
	ScpC	C5a (predicted)	Predicted cell surface	[36]

*Streptococcus zooepidemicus*	SzoCEP or ScpC	CXCL8	Cell surface and released	[52]
	
	ScpZ	C5a (predicted)	Predicted cell surface	[35]

*Streptococcus iniae*	ScpI	C5a	Cell surface and released	[37]
	
	CepI	CXCL8	Cell surface and released	[59]

CEPs with characterized function have been included, but it must be noted that there is an abundance of S8 protease sequences that have been inferred from homology with SpyCEP and ScpA/ScpB, which require experimental confirmation. CEP, cell-envelope protease; SpyCEP, *Streptococcus pyogenes* CEP.

**Table 2:** Percentage sequence identity between functionally characterized CEPs

CspA	−								
CepI	42%	−							
SpyCEP	39%	56%	−						
SeCEP	39%	56%	61%	−					
SzoCEP	39%	56%	62%	96%	−				
ScpC	32%	29%	30%	29%	29%	−			
ScpZ	31%	29%	29%	29%	29%	95%	−		
ScpI	32%	31%	32%	31%	31%	32%	32%	−	
ScpA	34%	33%	34%	34%	33%	38%	38%	38%	−
ScpB	33%	31%	32%	31%	31%	36%	36%	37%	98% −

CEP, cell-envelope protease; SpyCEP, *Streptococcus pyogenes* CEP.

**Table 3:** Summary of studies using CEPs as vaccine antigens for immunization against streptococcal species

Target	Vaccine	Model	Findings	Reference
SpyCEP	Recombinant SpyCEP with alum adjuvant	IP immunisation with M1 GAS IN challenge	Increased survival after challenge	[88]
	
	Recombinant SpyCEP with Freund's adjuvant	IP immunization followed by intranasal lethal challenge with M23 GAS of CD1 mice	Increased survival to 70%	[99]
	
	Recombinant SpyCEP with Freund's adjuvant	BALB/c mice immunized IM, followed by IM and IN challenge with GAS and *S. equi*	No reduction in bacterial burden at the site of infection. Decreased dissemination of GAS and *S. equi* following both IM and IN challenge	[52]
	
	Combo (SpyCEP, SpyAD, and streptolysin O) adjuvanted with Freund's or alum hydroxide	CD1 mice immunized IP, challenged with Ml, M6, M12, and M23 GAS	Cross serotype protection against GAS 50-80% survival following IN and IM challenge Decreased bacterial burden in subcutaneous air pouch infection Induced antibodies that mediated opsonophagocytic killing in whole blood assay	[101]
	
	SpyCEP with Freund's adjuvant	FVB/n mice immunized IM and IN challenge	Reduced signal of bioluminescent M75 GAS, but no difference in bacterial burden in the nasopharynx	[112]
	
	SpyCEP with J8-DT (M protein conserved epitope)	BALB/c mice immunized SC, GAS skin challenge following scarification	Protection against pyoderma and bacteraemia Protection against GAS hypervirulent CovRS mutants	[105, 106]

ScpA	Recombinant ScpA unadjuvanted	Intranasal immunization of CD1 mice, intranasal challenge with M1, M2, M6, M11, and M49 GAS	Reduced nasopharyngeal colonization	[24]
	
	Recombinant ScpA adjuvanted with alum or monophosphoryl lipid A	SC immunization of CD1 mice, IN challenge with M49 and M1 GAS	ScpA-specific antibodies neutralized protease activity of ScpA and ScpB Increased clearance of GAS from lungs and nasal mucosa	[109]
	
	ScpA adjuvanted with cholera toxin	Intranasal immunization of BALB/c mice, intranasal challenge with M49 GAS	Reduction of colonization of nasal-associated lymphoid tissue Immunized sera gave passive protection against GAS IN infection in naïve mice	[110]

SpyCEP and ScpA	Spy7 (ScpA, and 6 other highly conserved surface antigens) with Freund's adjuvant vs SpyCEP	FVB/n mice immunized IM, IM challenge with M1 and M3 GAS	Reduction in systemic dissemination	[102]
	
	Combo 5 (ScpA, SpyCEP, arginine deaminase, streptolysin O, and trigger factor) with Alum adjuvant	IM immunization of rhesus macaques, M1 GAS IN challenge	Robust antigen-specific antibody response and reduction in both pharyngitis and tonsillitis following an M1 intranasal challenge	[100]

ScpB	ScpB adjuvanted with alum and mycobacterial phospholipid, SC immunisation	CD1 mice, GBS serotype VI IN challenge	Reduced lung bacterial burden Conjugation of recombinant ScpB to a nonhomologous polysaccharide (III) can increase the immunogenicity of the polysaccharide when challenged with another serotype (VI) and reduced inflammatory damage to the lungs	[111]
	
	ScpB encapsulated in biodegradable polymer	ICR mice immunized IN or IM, vaginal challenge	Strong IgG response after administration of ScpB by both intranasal and intramuscular routes, with an increased response when encapsulated Intranasal administration elicited a secretory IgA in the vaginal mucosa Mice immunized with encapsulated ScpB fully resisted vaginal colonization after exposure to serotype III GBS. Parental immunization conferred neonate protection	[114]

SspA	SspA with ScpCL and 3 other *S. suis* virulence factors adjuvanted with CpG	IN immunization of C57BL6/6JCnc mice, followed by IN, IP, or IV challenge with SS2, SS3, and SS7 serotype *S. suis*	Significantly reduced NALT bacterial burden Serotype-dependent protection against systemic dissemination Reduced mortality in mice given a lethal IV dose of SS2 *S. suis*	[120]

PrtA	PrtA with Freund's adjuvant	C3h/HeJ mice, SC immunisation and IP challenge	Mice were protected from lethal challenge of serotypes 6A and 6B but not 4 *S. pneumoniae* strains Serum from immunized mice was able to protect naive mice from lethal challenge in a serotype- specific manner	[121]
	
	PrtA with ISCOMATRIX	IM immunization of BALB/c and CBA/n mice, followed by IN challenge with serotype 3 or 8 *S. pneumoniae*	Reduced serotype 3 bacterial burden in the lungs of BALB/c mice Protected CBA/n mice from a lethal intranasal dose of serotype 3 Protective effect against non-lethal serotype 8 infection	[122]
	
	PrtA with Curdlan adjuvant	IN immunization of BALB/c mice, followed by IN challenge with serotype 2 *S. pneumoniae*	Increased PrtA-specific IgG and IgA levels in BALF and increased IgA levels in saliva and nasal washes Did not protect against acute pneumonia and systemic dissemination; no significant reduction in lung bacterial burden or blood multiplication	[123]

CEPs, cell-envelope proteases; GAS, group A streptococcus; SpyCEP, *Streptococcus pyogenes* CEP.
